# A novel self-microemulsifying formulation of paclitaxel for oral administration to patients with advanced cancer

**DOI:** 10.1038/sj.bjc.6603312

**Published:** 2006-08-22

**Authors:** S A Veltkamp, B Thijssen, J S Garrigue, G Lambert, F Lallemand, F Binlich, A D R Huitema, B Nuijen, A Nol, J H Beijnen, J H M Schellens

**Affiliations:** 1 Division of Experimental Therapy, The Netherlands Cancer Institute/Antoni van Leeuwenhoek Hospital, 1066 CX Amsterdam, The Netherlands; 2 Department of Medical Oncology, The Netherlands Cancer Institute/Antoni van Leeuwenhoek Hospital, 1066 CX Amsterdam, The Netherlands; 3 Department of Pharmacy and Pharmacology, The Netherlands Cancer Institute/Slotervaart Hospital, 1066 EC Amsterdam, The Netherlands; 4 Novagali Pharma SA, 91058 Evry cedex, France; 5 Division of Biomedical Analysis, Faculty of Pharmaceutical Sciences, University of Utrecht, 3584 CG Utrecht, The Netherlands

**Keywords:** paclitaxel, cremophor-free, formulation, pharmacokinetics, safety

## Abstract

To explore the parmacokinetics, safety and tolerability of paclitaxel after oral administration of SMEOF#3, a novel Self-Microemulsifying Oily Formulation, in combination with cyclosporin A (CsA) in patients with advanced cancer. Seven patients were enrolled and randomly assigned to receive oral paclitaxel (SMEOF#3) 160 mg+CsA 700 mg on day 1, followed by oral paclitaxel (Taxol®) 160 mg+CsA 700 mg on day 8 (group I) or vice versa (group II). Patients received paclitaxel (Taxol®) 160 mg as 3-h infusion on day 15. The median (range) area under the plasma concentration–time curve of paclitaxel was 2.06 (1.15–3.47) *μ*g h ml^−1^ and 1.97 (0.58–3.22) *μ*g h ml^−1^ after oral administration of SMEOF#3 and Taxol®, respectively, and 4.69 (3.90–6.09) *μ*g h ml^−1^ after intravenous Taxol®. Oral SMEOF#3 resulted in a lower median *T*_max_ of 2.0 (0.5–2.0) h than orally applied Taxol® (*T*_max_=4.0 (0.8–6.1) h, *P*=0.02). The median apparent bioavailability of paclitaxel was 40 (19–83)% and 55 (9–70)% for the oral SMEOF#3 and oral Taxol® formulation, respectively. Oral paclitaxel administered as SMEOF#3 or Taxol® was safe and well tolerated by the patients. Remarkably, the SMEOF#3 formulation resulted in a significantly lower *T*_max_ than orally applied Taxol®, probably due to the excipients in the SMEOF#3 formulation resulting in a higher absorption rate of paclitaxel.

Currently, paclitaxel is only marketed as an intravenous (i.v.) formulation. Paclitaxel is poorly soluble in most pharmaceutical solvents, therefore, in the marketed i.v. formulation it is formulated in a 1:1 combination of the solubilising agent polyoxyethylated caster oil (Cremophor® EL (CrEL)) and dehydrated ethanol. CrEL has been reported to be responsible for severe hypersensitivity reactions (Webster *et al*, 1997) and the nonlinear pharmacokinetic behaviour of i.v. administered paclitaxel (Sparreboom *et al*, 1996; van Tellingen *et al*, 1999; van Zuylen *et al*, 2001a).

Oral administration of paclitaxel might be attractive because it is more convenient for patients than i.v. administration. Furthermore, oral paclitaxel administration may enable the development of treatment regimens resulting in plasma concentrations above a pharmacologically relevant level for more prolonged periods of time. However, oral treatment with paclitaxel is severely hampered because of its low bioavailability, which is caused by two main reasons. Firstly, paclitaxel is a high-affinity substrate for the efflux multidrug transporter P-glycoprotein (P-gp), which is highly expressed in the gastrointestinal tract (Sparreboom *et al*, 1997). Secondly, paclitaxel undergoes first-pass metabolism by the gut and liver cytochrome P450 (CYP) enzymes (CYP 2C8 and CYP 3A4).

Previous studies carried out at our Institute investigated the pharmacokinetics of the i.v. paclitaxel formulation after oral administration as a drinking solution diluted with water (Meerum Terwogt *et al*, 1999; Malingré *et al*, 2000a, 2000b; Malingré *et al*, 2001a, 2001b, 2001c, 2001d, 2001e) and revealed that coadministration of oral CsA resulted in an increased systemic exposure to oral paclitaxel (van Asperen *et al*, 1998; Meerum Terwogt *et al*, 1999). As CsA is an inhibitor of both P-gp and CYP3A4, both an increased absorption and a reduced first-pass effect may be responsible for the increased systemic exposure. We have shown previously that systemic exposure to paclitaxel did not increase with CsA doses higher than 10 mg kg^−1^ (Malingré *et al*, 2001a, 2001b, 2001c, 2001d, 2001e).

Although CrEL was reported to exhibit no oral absorption (Malingré *et al*, 2001a, 2001b, 2001c, 2001d, 2001e) it affects paclitaxel pharmacokinetics by limiting the absorption of paclitaxel from the intestine after oral administration, probably by entrapment of paclitaxel in micelles, thereby reducing the availability of paclitaxel for uptake (Sparreboom *et al*, 1996; Sparreboom *et al*, 1999; Malingré *et al*, 2001a, 2001b, 2001c, 2001d, 2001e; van Zuylen *et al*, 2001b; Bardelmeijer *et al*, 2002). Thus far, a favourable oral formulation with paclitaxel has not been found yet.

Self-Microemulsifying Oily Formulation (SMEOF)#3 is a novel oral SMEOF of paclitaxel. The formulation consists of an isotropic mixture of oils and surfactants, which solubilise paclitaxel and spontaneously forms a microemulsion upon contact with water. Previous *in vivo* studies in *wild-type* mice and *mdr1a/b (*−/−) knockout mice, which lack functional P-gp activity, showed that SMEOF#3 was a suitable delivery vehicle for oral administration of paclitaxel in combination with oral CsA (data on file). The choice of the excipients was motivated by a previous study exploring particle size, physical and chemical stability as well as cytotoxic activity *in vitro* of different formulations of paclitaxel (Gursoy *et al*, 2003). Furthermore, the physical stability was assessed of emulsions of different SMEOFs of paclitaxel after dilution in simulated gastric fluid (SGF). It was shown that after dilution of the i.v. paclitaxel (Taxol®) formulation in SGF, major part of the micelles was destabilised and a massive precipitation immediately occurred (data on file). This phenomenon was thought to be due to diffusion of ethanol in the SGF after which the remaining CrEL was not capable to maintain all paclitaxel in solution. SMEOF#3, however, showed a good stability for about 6 h after dilution in SGF. In SMEOF#3, tyloxapol and TPGS (d-alpha-tocopheryl polyethylene glycol 1000 succinate) have been selected for their ability to solubilise paclitaxel. Tyloxapol is a nonionic surfactant used in the product Exosurf® (GlaxoSmithKline, UK). Upon contact with water, tyloxapol was shown to form lyotropic liquid crystals, which are are thought to contribute to the formation of microemulsions (Gershanik and Benita, 2000). Furthermore, it was shown that tyloxapol can be used as a cosolvent by increasing the solubility of paclitaxel in ethanol (Gursoy *et al*, 2003). TPGS is a derivative of vitamin E with amphiphillic properties and it is used as excipient in Agenerase® (amprenavir, GlaxoSmithKline, UK). TPGS has been shown to increase the bioavailability of poorly absorbed lipophilic drugs (Chang *et al*, 1996), probably by its solubilising effect through improved micelle formation (Boudreaux *et al*, 1993). As compared to the commercial paclitaxel (Taxol®) formulation tested orally, the main advantages of SMEOF#3 are the absence of CrEL and the significantly lower ethanol/paclitaxel ratio, which allows reducing the amount of ethanol to be administered.

The purpose of this study was to investigate the pharmacokinetics, safety and tolerability of this novel oral formulation of paclitaxel (SMEOF#3).

## PATIENTS AND METHODS

### Patient population

Patients with a histological or cytological diagnosis of advanced nonhaematological cancer for whom no curative therapy existed and for whom treatment with single agent paclitaxel was considered of potential benefit were eligible for the study. Patients had to be recovered from any toxicities of prior treatment. Previous chemotherapy was allowed as long as the last treatment was at least 4 weeks prior to study entry and at least 3 weeks should have elapsed since receiving radiotherapy.

Patients had to have acceptable haematological parameters (white blood cells (WBC) ⩾3.0 × 10^9^ l^−1^, and platelets ⩾100 × 10^9^ l^−1^)), hepatic function (serum bilirubin ⩽20 *μ*mol l^−1^; AST and ALT⩽1.5 × upper limit of normal (ULN) or ⩽3 × ULN in case of liver metastases) and renal function (serum creatinine ⩽160 *μ*mol l^−1^ or creatinine clearance ⩾50 ml min^−1^ as calculated by Cockcroft Gault formula), and a World Health Organization (WHO) performance status (PS) ⩽2. Patients were excluded if they had experienced severe toxicities on prior taxane treatment, suffered from uncontrolled infectious disease, heart disease, bowel obstruction or motility disorders that could have influenced the resorption of drugs. Further exclusion criteria were concomitant use of known P-gp and CYP 3A modulating drugs and chronic use of H2-receptor antagonists or proton pump inhibitors. Female patients were excluded when breast-feeding or pregnant (confirmed by a pregnancy test before study entry). Patients had to be willing and able to follow the protocol requirements. The Medical Ethics Committee of the Institute approved the study protocol and all patients gave written informed consent.

### Study design

Initially six patients were planned to be enrolled in the study and were randomly assigned to two groups of treatment. Group I received a combination of oral paclitaxel (SMEOF#3) 160 mg and CsA 700 mg on day 1, followed by oral paclitaxel (Taxol®) 160 mg in combination with CsA 700 mg on day 8, and group II received oral paclitaxel (Taxol®) 160 mg in combination with CsA 700 mg on day 1, followed by oral paclitaxel (SMEOF#3) 160 mg and CsA 700 mg on day 8. CsA was administered orally at a fixed dose of 700 mg (approximately equivalent to 10 mg kg^−1^ CsA) 30 min prior to oral administration of paclitaxel. The patients in both groups received a single i.v. administration of paclitaxel (Taxol®) 160 mg as a 3-h infusion on day 15.

### Drug composition and administration

The composition of SMEOF#3 (Novagali Pharma SA, Evry cedex, France) is presented in Table 1. SMEOF#3 (160 mg in 10 ml) was administered orally to the patients via a syringe within 30 min after 1:3 dilution with tap water to 40 ml resulting in a final paclitaxel concentration of 4 mg ml^−1^. The commercially available i.v. paclitaxel (Taxol®) formulation (Bristol-Myers Squibb, Syracuse, NY, USA) containing paclitaxel 6 mg ml^−1^, ethanol 396 mg ml^−1^, and CrEL 527 mg ml^−1^ was administered orally after dilution of 26.7 ml (160 mg) with water to 40 ml (4 mg ml^−1^). The i.v. paclitaxel (Taxol®) formulation was administered i.v. at a fixed dose of 160 mg as 3-h infusion to all patients on day 15. CsA was administered as seven capsules of 100 mg each (Neoral®, Novartis, Basel, Switzerland).

To prevent possible nausea and vomiting during both the treatment with oral SMEOF#3 and oral Taxol®, patients were premedicated with oral granisetron (Kytril®) 1 mg approximately 2 h before the intake of paclitaxel. In addition, patients received a light standard breakfast (two crackers and a cup of tea) at least 2 h prior to each paclitaxel administration. Intake of food was not allowed until 2 h following the intake of oral paclitaxel. Patients were premedicated with dexamethasone 20 mg p.o. 8–10 h prior to, and ranitidine 50 mg i.v., clemastine 2 mg i.v. and dexamethasone 20 mg i.v., 30–60 min before i.v. paclitaxel dosing, to prevent infusion-related hypersensitivity reactions. If considered in their best interest, patients continued on a 3-weekly schedule of i.v. paclitaxel at a dose of 175 mg m^−2^.

### Sample collection and analysis

Blood samples for pharmacokinetic analysis of paclitaxel were collected via an indwelling catheter in 5 ml heparinised tubes after both p.o. and i.v. administration. Following oral administration samples were obtained prior to administration, immediately after administration, and 15, 30, 45 min, and 1, 2, 4, 6, 8, 24, 48, and 72 h after paclitaxel administration. Following i.v. administration samples were obtained prior to administration, 60, 120, and 165 min after start of infusion, at the end of infusion, and 15, 30, 45 min, and 1, 2, 4, 6, 8, 24, 48, and 72 h after infusion. Blood samples were centrifuged, and plasma was separated and immediately transferred into polypropylene tubes and stored at −20°C until analysis. Paclitaxel concentrations in plasma were determined using a validated high performance liquid chromatographic (HPLC) tandem mass spectrometric (MS/MS) method (Vainchtein *et al*, 2006).

For the pharmacokinetic analysis of CsA, blood samples were collected in 5 ml EDTA tubes at the same time points as for paclitaxel after the oral SMEOF#3 and oral Taxol® administrations. Whole blood samples were stored at 4°C until analysis using a specific fluorescence polarisation immunoassay (FPIA) (Malingré, 2001).

Urine samples were collected prior to paclitaxel administration and at the intervals: 0–24, 24–48, and 48–72 h after oral SMEOF#3 and oral Taxol® and after i.v. Taxol® administration. A volume of 19 ml of each urine sample was mixed with 1 ml of a mixture of 5% CrEL (Sigma, Prague, Czech Republic) – ethanol (Merck, Darmstadt, Germany) 1:1 v v^−1^ to prevent paclitaxel precipitation. Subsequently, 5 ml was transferred into a 10 ml polypropylene tube and stored at −20°C until analysis. Paclitaxel concentrations in urine were determined using a validated HPLC method with ultraviolet (UV) detection (Huizing *et al*, 1995).

### Pharmacokinetics

The pharmacokinetic parameters of paclitaxel and CsA were determined by noncompartmental analysis, using WinNonLin™ (version 5.0, Pharsight Corporation, Mountain View, CA, USA). The area under the plasma concentration time curve (AUC) was determined using the linear logarithmic trapezoidal method up to the last measured concentration-time point and extrapolated to infinity (AUC_0−∞_) using the slope of the terminal part of the logarithmic concentration *vs* time curve (*λ*_z_). Furthermore, the terminal half-life (*t*_1/2_) was determined. The maximal observed drug concentration (*C*_max_) and time to maximal observed drug concentration (*T*_max_) were obtained directly from the experimental data.

The apparent bioavailability (F) of paclitaxel was calculated by the ratio of the AUC_0−∞_ after oral administration and AUC_0−∞_ after i.v. administration of paclitaxel. Furthermore, the fraction of the paclitaxel dose that was excreted unchanged in urine was calculated.

### Statistics

The software package Statistical Product and Service Solutions (version 12.1. 1 for Windows, SPSS Inc., Chicago, IL, USA) was used for statistical analysis. The priori level of significance was *P*=0.05. The paired *t*-test was applied on logarithmic-transformed values to make a comparison between the pharmacokinetic parameters of paclitaxel after the different study treatments.

### Safety

All toxicities observed were graded according to the National Cancer Institute Common Terminology Criteria for Adverse Events (NCI CTCAE) version 3.0, 2003 (http://ctep.cancer.gov/forms/CTCAEv3.pdf).

## RESULTS

### Patient characteristics

As one patient was not fully evaluable for pharmacokinetic analysis, one additional patient was included and in total seven patients were entered into the study. Patient characteristics are specified in Table 2. Patients 1, 4, 5, and 7 were assigned to group I and patients 2, 3, and 6 were assigned to group II. Four patients and three patients had a PS of 1 and 2, respectively. Age, height, and weight appeared to be equally distributed over the two treatment groups.

### Drug administration and extent of exposure

All patients received all three treatments (day 1, 8, and 15) at the single flat dose of 160 mg per formulation. The i.v. administration of Taxol® during day 15 was temporally interrupted in patient 1 due to infusion leakage. Patient 4, a 54 years old female, developed rash and dyspnoea 15 min following i.v. paclitaxel administration. The paclitaxel infusion was terminated and 2 mg clemastine i.v. was given, which resolved the hypersensitivity reaction. Therefore, blood sampling for pharmacokinetics could not be performed and this patient was not fully eligible. Intravenous paclitaxel administration was restarted after one hour at a lower infusion rate, which did not cause any adverse reactions.

### Pharmacokinetic and statistical analysis

**Figure 1:**
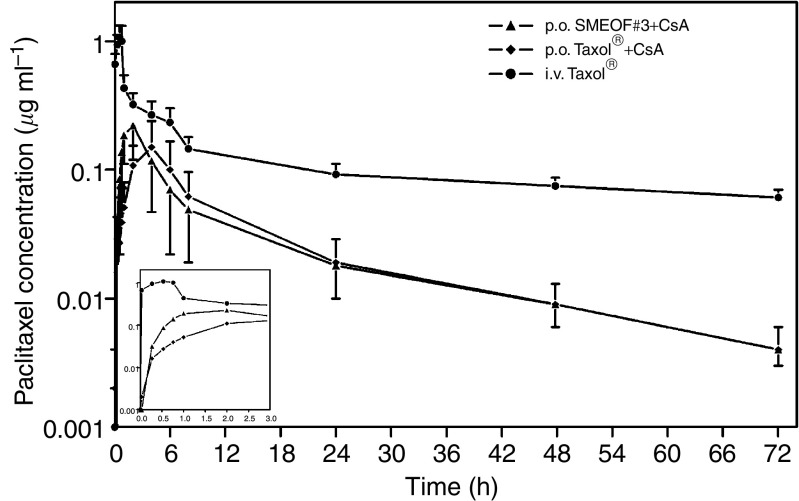
Paclitaxel plasma concentration *vs* time curves after p.o. SMEOF#3 160 mg+CsA 700 mg, p.o. Taxol® 160 mg+CsA 700 mg (*n*=7), and i.v. Taxol® 160 mg as 3 h infusion (*n*=6). Data are represented as mean±s.d. on a semi-logarithmic scale.

Figure 1 depicts the plasma pharmacokinetic profiles of paclitaxel after treatment with p.o. paclitaxel (SMEOF#3), p.o. paclitaxel (Taxol®) (*n*=7), and i.v. paclitaxel (Taxol®) 160 mg as 3 h infusion (*n*=6). Interpatient variability in paclitaxel plasma concentrations was comparable between p.o. SMEOF#3 and p.o. Taxol®, both coadministered with CsA, but was lower after i.v. administered Taxol®.

**Figure 2:**
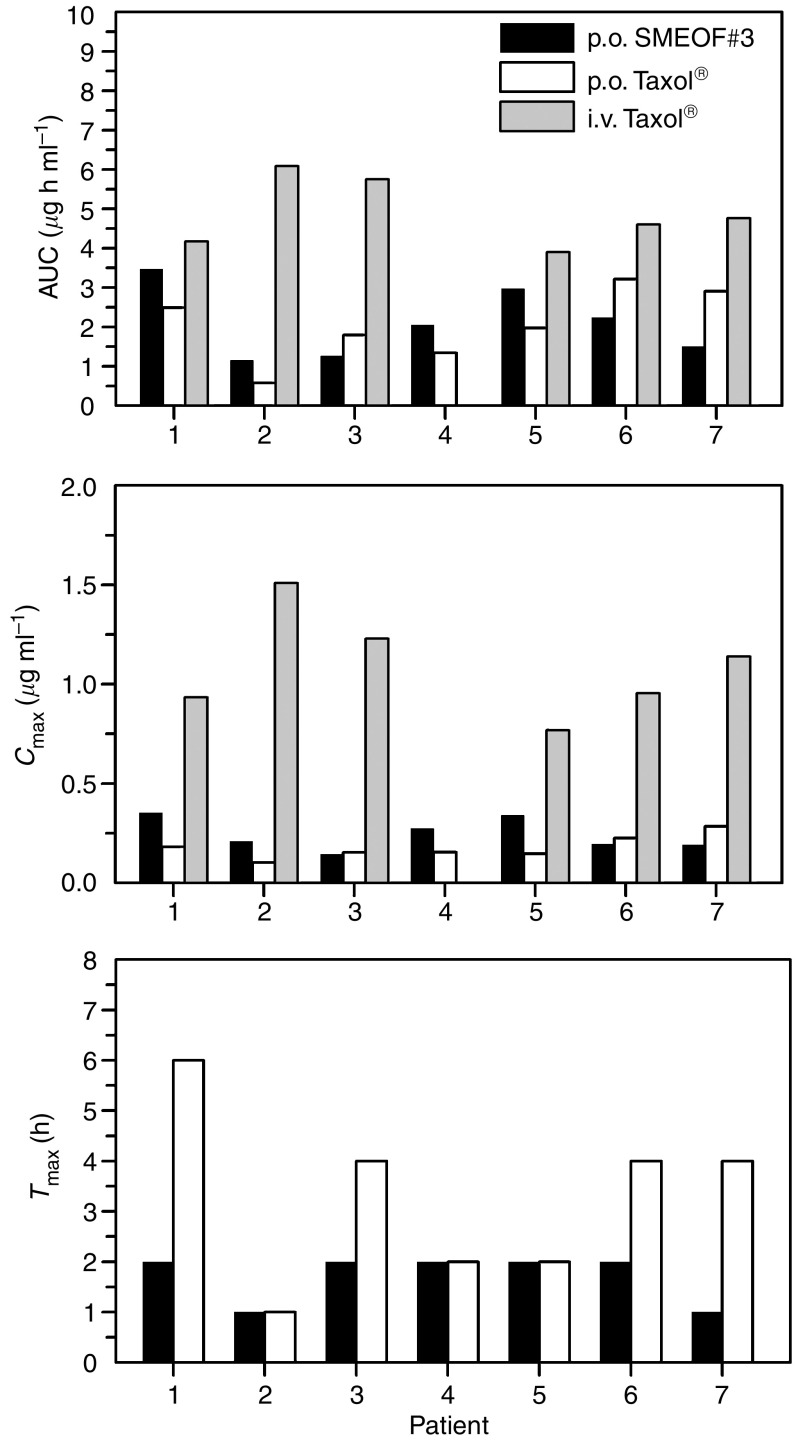
Individual AUC_0−∞_, *C*_max_, and *T*_max_ values of paclitaxel after treatment with p.o. SMEOF#3 and p.o. Taxol® (*n*=7), both coadministered with CsA, and after treatment with i.v. Taxol® (*n*=6).

Figure 2 presents the AUC_0−∞_ (*μ*g h ml^−1^), *C*_max_ (*μ*g ml^−1^), and *T*_max_ (h) of paclitaxel after p.o. SMEOF#3, p.o. Taxol® and after i.v. Taxol® given as 3 h infusion. *T*_max_ after oral SMEOF#3 was substantially lower compared to p.o. paclitaxel (Taxol®) (*P*=0.021).

The plasma pharmacokinetic parameters of paclitaxel after the three study treatments are depicted in Table 3. The median (range) AUC_0−∞_ of the SMEOF#3 formulation was 2.06 (1.15–3.47) *μ*g h ml^−1^, which was not significantly higher than the AUC_0−∞_ of 1.97 (0.58–3.22) *μ*g h ml^−1^ after oral Taxol® (*P*=0.74). The interpatient variability in AUC_0−∞_ was relatively high after both p.o. SMEOF#3 (%CV=42) and p.o. Taxol® (%CV=45). Furthermore, oral SMEOF#3 resulted in a not significantly higher median *C*_max_ of 0.21 (0.15–0.35) *μ*g ml^−1^ compared to a *C*_max_ of 0.16 (0.10–0.29) *μ*g ml^−1^ after oral Taxol® (*P*=0.15). Remarkably, oral SMEOF#3 showed a significantly lower *T*_max_ of 2.0 (0.5–2.0) h than p.o. Taxol®, which had a *T*_max_ of 4.0 (0.8–6.1) h (*P*=0.021). The median apparent bioavailability was 40% (19–83%) and 55% (9–70%) for the oral SMEOF#3 and oral Taxol® formulation, respectively. After both i.v. and oral administration excretion of paclitaxel in the urine was low and more than 70% of the total urinary excretion occurred within 24 h.

**Figure 3:**
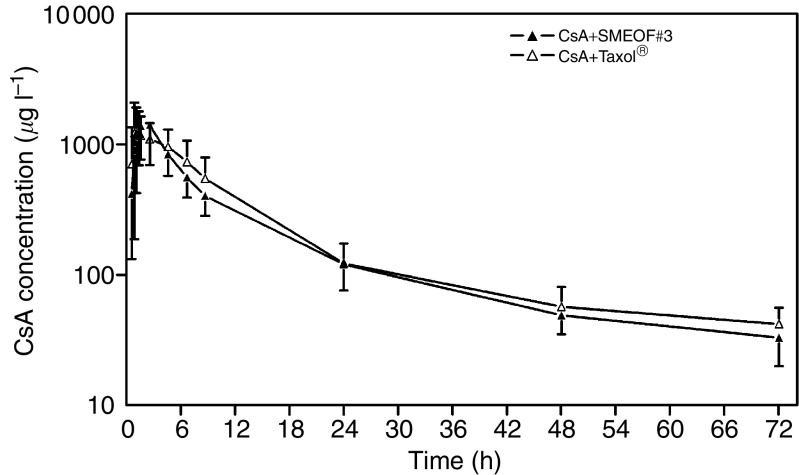
CsA blood concentration *vs* time curves after administration of CsA 700 mg in combination with oral SMEOF#3 and oral Taxol® (*n*=7). Data are represented as mean±s.d. on a semi-logarithmic scale.

Figure 3 depicts the mean pharmacokinetic profiles of CsA after oral administration of CsA 700 mg in combination with p.o. SMEOF3# 160 mg and p.o. Taxol® 160 mg (*n*=7). The pharmacokinetic parameters of CsA of all three study treatments are summarised in Table 4.

Figure 3 and Table 4 clearly show that CsA pharmacokinetics were not influenced by coadministration of either paclitaxel formulations.

### Safety evaluation

Nonhaematological toxicities were CTCAE grade 1–2, except for two grade 3 events: one hypersensitivity reaction in patient 4 after i.v. paclitaxel administration, and muscular weakness in patient 1, that was considered to be probably related to SMEOF#3. No life threatening adverse events (grade 4) and deaths (grade 5) were reported in the study. Overall, the most frequently reported drug-related adverse events were gastrointestinal disorders with the most common symptoms of nausea occurring in three patients after oral administration of paclitaxel. Furthermore, abdominal pain, diarrhoea, and stomatitis were reported in two patients. No clinical relevant haematological toxicities occurred after the three treatments. Furthermore, no abnormal blood chemistry values were reported.

## DISCUSSION

In the present clinical study, we tested the pharmacokinetics, safety and tolerability of SMEOF#3, a new micro-emulsifying formulation for oral administration of paclitaxel in combination with CsA.

The apparent bioavailability of paclitaxel after oral administration of SMEOF#3 coadministered with CsA was estimated at 40% (19–83%) and was comparable to the apparent bioavailability of orally administered Taxol®. These data were in line with studies that showed that the apparent bioavailability of orally administered Taxol® in combination with CsA was approximately 47% (Huizing *et al*, 1997; Meerum Terwogt *et al*, 1999).

The term bioavailability, however, should be interpreted with caution due to the nonlinear pharmacokinetics of i.v. paclitaxel caused by the presence of CrEL (van Tellingen *et al*, 1999). Entrapment of paclitaxel in CrEL micelles in the central compartment causes a more than proportional increase in plasma paclitaxel concentrations with increasing doses. Studies in mice showed that these higher total drug levels in plasma did not result in higher drug levels in tissues (Sparreboom *et al*, 1996). Previous studies showed that CrEL is not absorbed after oral administration. This pseudo-nonlinearity of i.v. paclitaxel has two important implications for the pharmacology of oral paclitaxel. Firstly, the oral bioavailability of paclitaxel, calculated by comparing the AUC values after oral and i.v. administration, will be underestimated as the affinity of paclitaxel for the plasma compartment is increased after i.v. administration due to the presence of CrEL in the central circulation. Secondly, the pseudo-nonlinearity of i.v. paclitaxel implies that after oral administration, when CrEL is not present, plasma levels of paclitaxel represent a higher fraction of free drug, which will result in enhancement of the availability of paclitaxel for the (tumour) tissues (van Tellingen *et al*, 1999). Consequently, threshold values for the paclitaxel concentration established for i.v. paclitaxel (Gianni *et al*, 1995; Huizing *et al*, 1997) cannot be used for oral administration of paclitaxel.

The pharmacokinetic parameters of CsA after coadministration with oral SMEOF#3 and orally administered Taxol® were comparable. Furthermore, pharmacokinetic parameters of CsA were in line with those obtained before (Malingré *et al*, 2001a, 2001b, 2001c, 2001d, 2001e). It has been demonstrated that 10 mg kg^−1^ CsA was sufficient for maximal enhancement of paclitaxel bioavailability (Malingré *et al*, 2001a, 2001b, 2001c, 2001d, 2001e). In combination, these facts suggest that a dose of 700 mg CsA as used in this study was sufficient.

Remarkably, the *T*_max_ of paclitaxel after oral administration of the SMEOF#3 formulation was lower compared to oral Taxol®. It was previously described by us that CrEL limits the absorption rate of paclitaxel due to encapsulation in CrEL micelles. As the concentration of CrEL in the gastrointestinal tract decreases with time due to distribution, breakdown and elimination of CrEL, more unbound paclitaxel becomes available for absorption in the systemic circulation with time and consequently the absorption rate increases (de Jonge *et al*, 2005). The lower *T*_max_ after oral SMEOF#3 is probably due to the ability of the SMEOF#3 formulation to remain stable in the gastrointestinal tract avoiding precipitation of paclitaxel leading to a major fraction of paclitaxel in solution, which is available for absorption. However, in the case of oral paclitaxel administered as Taxol®, probably a significant amount of paclitaxel precipitates due to quick diffusion and resorption of ethanol and the precipitated fraction of paclitaxel is slowly re-dissolved in the gastrointestinal fluids before being absorbed. Furthermore, a lower amount of ethanol was administered after SMEOF#3 160 mg compared to the orally applied i.v. paclitaxel (Taxol®) formulation 160 mg; the amount of administered ethanol was approximately 3.3 g and 10.6 g after SMEOF#3 and the orally applied i.v. paclitaxel (Taxol®) formulation, respectively.

An extensive pharmacokinetic analysis demonstrated an increase in the systemic exposure to paclitaxel and a prolonged time of a paclitaxel concentration above a pharmacological relevant level with increasing doses of SMEOF#3 (data not shown).

In summary, we demonstrated that the novel SMEOF#3 formulation was well tolerated after oral administration at the given dose of 160 mg when coadministered with CsA, without induction of relevant gastrointestinal or haematological toxicity. Regarding the nearly uneventful oral administration of the 160 mg dose together with the relatively low AUC_0−∞_ after CsA coadministration, we suggest that new studies should be initiated with this novel SMEOF#3 formulation to explore once daily administration of paclitaxel at higher dose levels in order to increase systemic exposure and to prolong exposure at therapeutic levels.

## Figures and Tables

**Table 1:** Composition of SMEOF#3

**Component**	**% (w v^−1^)**	**Function**
Paclitaxel	1.6	Active substance
Vitamin E	5	Oil
TPGS^a^	30	Surfactant, cosolvent
Tyloxapol	30	Surfactant, cosolvent
Ethanol (anhydrous)	33.38	Solvent
Citric acid (anhydrous)	0.02	pH adjuster
Total	100	

a D alpha-tocopheryl polyethylene glycol 1000 succinate.

**Table 2:** Patient characteristics

No. of patients	7
Male/female	5/2
Median age, years (range)	56 (29–63)
Median PS (range)	1 (1–2)
	
Tumour type	
NSCL	1
Stomach	1
Neuroendocrinal	1
Thyroid	1
Oesophagus cardia	2
Parotid gland	1
	
Prior treatment	
Surgical therapy	5
Chemotherapy	7
Radiotherapy	4

**Table 3:** Plasma pharmacokinetic parameters of paclitaxel after p.o. SMEOF#3 160 mg, p.o. Taxol® 160 mg (*n*=7), and i.v. Taxol® 160 mg as 3 h infusion (*n*=6)

**Parameter**	**SMEOF#3 p.o.**	**Taxol® p.o.**	**Taxol® i.v.**
*T*_max_ (h)	2.0 (0.5–2.0)	4.0 (0.8–6.1)	NA
*C*_max_ (*μ*g ml^−1^)	0.21 (0.15–0.35)	0.16 (0.10–0.29)	1.05 (0.77–0.15)
AUC_0-∞_ (*μ*g h ml^−1^)	2.06 (1.15–3.47)	1.97 (0.58–3.22)	4.69 (3.90–6.09)
%CV of AUC	42	45	18
*t*_1/2_ (h)	23 (20–28)	22 (17–33)	23 (16–32)
F(%)	40 (19–83)	55 (9–70)	
%CV of F	59	48	
*U*_excr_ (% of dose)^a^	1.3 (0.5–2.1)	1.7 (0.6–3.6)	5.0 (3.4–8.3)

Data are presented as median (range).

NA, not applicable.

a *U*_excr_, urinary paclitaxel excretion.

%CV % coefficient of variation.

**Table 4:** Plasma pharmacokinetic parameters of CsA after administration of CsA 700 mg in combination with p.o. SMEOF#3 160 mg and p.o. Taxol® 160 mg (*n*=7)

**Parameter**	**SMEOF#3**	**Taxol®**
*T*_max_ (h)	2.6 (0.8–2.8)	1.5 (0.8–6.7)
*C*_max_ (*μ*g ml^−1^)	1.48 (0.86–2.89)	1.51 (0.80–2.71)
AUC_0-∞_ (*μ*g h ml^−1^)	13.3 (10–23.8)	15.8 (8.95–25.1)
%CV of AUC	32	34
*t*_1/2_ (h)	17 (12–28)	20 (8–34)

Data are presented as median (range).
